# Arsenic nano complex induced degradation of YAP sensitized ESCC cancer cells to radiation and chemotherapy

**DOI:** 10.1186/s13578-020-00508-x

**Published:** 2020-12-22

**Authors:** Wei Zhou, Meiyue Liu, Xia Li, Peng Zhang, Jiong Li, Yue Zhao, Guogui Sun, Weimin Mao

**Affiliations:** 1 Cancer Hospital of University of Chinese Academy of Sciences (Zhejiang Cancer Hospital), Institute of Cancer and Basic Medicine of Chinese Academy of Sciences, Hangzhou, 310022 China; 2 School of Public Health, North China University of Science and Technology Affiliated People’s Hospital, North China University of Science and Technology, Tangshan, 063001 China; 3 Department of Medicinal Chemistry, Massey Cancer Center, Philips Institute for Oral Health Research , Virginia Commonwealth University, Richmond, VA 23298-0540 USA

**Keywords:** Arsenic nano complex, YAP, ESCC, ROS, Radiation therapy, Chemotherapy

## Abstract

**Background:**

Increased reactive oxygen species (ROS) production by arsenic treatment in solid tumors showed to be effective to sensitize cancer cells to chemotherapies. Arsenic nano compounds are known to increase the ROS production in solid tumors.

**Methods:**

In this study we developed arsenic–ferrosoferric oxide conjugated Nano Complex (As_2_S_2_–Fe_3_O_4,_ AFCNC) to further promote the ROS induction ability of arsenic reagent in solid tumors. We screen for the molecular pathways that are affect by arsenic treatment in ESCC cancer cells. And explored the underlying molecular mechanism for the arsenic mediated degradations of the key transcription factor we identified in the gene microarray screen. Mouse xenograft model were used to further verify the synthetic effects of AFCNC with chemo and radiation therapies, and the molecular target of arsenic treatment is verified with IHC analysis.

**Results:**

With gene expression microarray analysis we found Hippo signaling pathway is specifically affected by arsenic treatment, and induced ubiquitination mediated degradation of YAP in KYSE-450 esophageal squamous cell carcinoma (ESCC) cells. Mechanistically we proved PML physically interacted with YAP, and arsenic induced degradation PML mediated the degradation of YAP in ESCC cells. As a cancer stem cell related transcription factor, YAP 5SA over expressions in cancer cells are correlated with resistance to chemo and radiation therapies. We found AFCNC treatment inhibited the increased invasion and migration ability of YAP 5SA overexpressing KYSE-450 cells. AFCNC treatment also effectively reversed protective effects of YAP 5SA overexpression against cisplatin induced apoptosis in KYSE-450 cells. Lastly, with ESCC mouse xenograft model we found AFCNC combined with cisplatin treatment or radiation therapy significantly reduced the tumor volumes in vivo in the xenograft ESCC tumors.

**Conclusions:**

Together, these findings suggested besides ROS, YAP is a potential target for arsenic based therapy in ESCC, which should play an important role in the synthetic effects of arsenic nano complex with chemo and radiation therapy.

## Background

Arsenic compounds have been proven to be effective in the treatment of several types of cancers [1]. By targeting PML or PML-RARa for ubiquitination mediated degradation arsenic compounds successfully applied in the treatment of acute promyelocytic leukemia (APL) [2, 3]. The State Food and Drug Administration of China approved the use of arsenic trioxide for the treatment of advanced human primary hepatocellular carcinoma (HCC) in 2004 [4]. Several studies suggested arsenic compounds has other molecular targets in solid tumors [5, 6]. Wang et al. reported arsenic trioxide specifically inhibited transcriptional activity of SRF/MCM7 in liver cancer stem cells to inhibit metastasis of hepatocellular carcinoma [7]. Diepart et al. reported by inhibiting mitochondria respiration of the cancer cells, single dose of arsenic trioxide treatment induced upregulation of oxygenation and increased the sensitivity to radiation therapy of solid tumors [8]. Arsenic sulfide nano particle has been explored as a substitute for arsenic trioxide in the treatment of cancer due to less toxicity and improved bioavailability [6, 9, 10]. In this study we developed arsenic–ferrosoferric oxide (As_2_S_2_–Fe_3_O_4_) conjugated Nano Complex (AFCNC) to increase the ROS production efficacy of arsenic sulfide nano particle by promoting Fenton reactions [11].

Esophageal squamous cell carcinoma (ESCC) is the fourth leading cause of death among all the cancer patients in China, and a high incident cancer worldwide [12, 13]. We explored the molecular targets that are specifically affected by arsenic nano particle treatment in ESCC cancer cells with gene transcriptomic microarray analysis. We found Hippo signaling pathway is among the most affected pathways by arsenic treatment. Further molecular studies proved arsenic treatment induced ubiquitination mediated degradation of YAP protein in ESCC cancer cells.

YAP is a cancer stem cell related transcription factor that promotes tumor invasion and metastasis, and is correlated with poor prognosis and resistance to chemo and radiation therapy in ESCC cancer cells [14, 15]. We found combined administration of AFCNC with cisplatin or radiation therapy significantly inhibited tumor growth rates in the human ESCC mice xenograft model compared with cisplatin or radiation treatment alone. Together, our findings suggested arsenic–ferrosoferric oxide conjugated Nano Complex could be used in combination with chemo or radiation therapy to specifically target the cancer stem cell population of ESCC to inhibit treatments induced tumor dormancy and relapse [16].

## Materials and methods

Arsenic sulfide was purchased from Shanghai Yuanye Biotechnology, Ltd; FeCl_3_·6H_2_O and FeCl_2_·4H_2_O were purchased from Aladdin Chemical Reagent Company (Shanghai, China); 28% aqueous ammonia was purchased from Lingfeng Chemical Reagent Company (Shanghai, China). Zetasizer Nano Z90 Zeta potential analyzer (Malvern Analitical Ltd.); JEM-2100 Electron Microscope (JEOL Ltd.). MG-132 and YAP inhibitor Verteporfin was purchased from MCE Ltd. Co. ESCC cell lines KYSE-450, KYSE-30 were preserved in our laboratory. The ESCC cells were cultured in RPMI 1640 (Gibco) supplemented with 10% fetal bovine serum (FBS) at 37 °C under 5% CO_2_.

### Preparation of GSH (glutathione) embedded arsenic sulfide nano particle

Arsenic sulfide powder was purified by incubating with 0.2 mol/l hydrochloric acid for 12 h. The precipitation was wash with distillated water, and a laboratory dryer was used to dehydrate the purified arsenic sulfide. 16 g of Na_2_S was dissolved into 100 ml of distillated water, 30 g of purified arsenic sulfide was added to the sodium sulfide solution and blended for at least 4 h at 35 ℃, then discard unresolved arsenic sulfide precipitate, and the PH of arsenic sulfide solution was adjusted to 7–8. 30 ml of the arsenic sulfide solution was mixed with 50 ml 5 mol/l glutathione solution, adjusted the pH value to 7–8 and incubated the mixed solution with a blender at 37 ℃ for 3 days to get the GSH embedded arsenic sulfide nanoparticles. Dialysis the solution for 3 days with distillated water in a ventilated camber, then preserve the GSH embedded arsenic sulfide nanoparticle at 4 ℃.

### Preparation of DMSA (dimercaptosuccinic acid) ferrosoferric oxide nano particle

20 ml distillated water was preincubated with argon gas for 20–30 min in a three-necked flask, the following procedures were all conducted in argon gas. 4 mmol FeCl_3_·6H_2_O and 2.4 mmol FeSO_4_·7H_2_O were added to the flask and blended for 10 min. Heated the solution to 60 ℃, then slowly added 2.5 ml ammonia solution in 1 min, and keep blending the solution for 30 min. Heated the solution to 80 ℃ and stop blending and let still the solution for 30 min, wash the precipitate with distillated water and ethanol several times before harvesting the precipitated ferrosoferric oxide nano particles. Dissolve 15 mg ferrosoferric oxide nano particles into 10 ml (2 mg/ml) dimercaptosuccinic acid, shaking the solution for 12 h and further incubating for 24 h. Wash the precipitate with distillated water for 3 times, then preserve the acquired ferrosoferric oxide nano in 10 ml distillated water.

### DCFH-DA cellular ROS detection assay

ROS production induced by different nano particles in KYSE-450 cells induced was evaluated with DCFH-DA cell-permeative probe. DCFH-DA (Dichloro-dihydro-fluorescein diacetate) is cleaved by cellular esterase and oxidized by ROS to yield green fluorescence in the cytoplasm. Briefly, ESCC KYSE-450 cells were seeded on Millicell EZ slides, and stained with 25 µM of DCFDA purchased from Sigma (D6883) for 40 min. The cells were washed with PBS for 3 times, and cultured back in RPMI-1640 full growth media and treated with different nano particles for 12 h. Cell nuclei were stained DAPI (1 µg/µl) for 10 min, then washed with PBS before fluorescence microscope observation with excitation/emission at 495 nm/529 nm and 358 nm/461 nm respectively. Fluorescent images were taken with Olympus Fluoview FV1200 microscope at the magnification of 40×.

### Gene microarray transcriptomic analysis

The gene microarray transcriptomic experiments of arsenic treated esophageal cancer cells were conducted by Shanghai OE Biotech Co., Ltd. Briefly, approximately three repeats of 1 × 10^7^ KYSE-450 ESCC cells were harvested, and total cellular RNAs were extracted, and the quantities and qualities of the RNAs were measured with NanoDrop ND-2000 (Thermo Scientific) and Agilent Bioanalyzer 2100 (Agilent Technologies). The total RNAs were reverse transcribed to cDNA libraries, and labelled with Cyanine-3-CTP (Cy3). The labelled cDNAs were hybridized with Agilent Human lncRNA Microarray 2018 (4*180 k, Design ID:085630), and scanned with Agilent Scanner G2505C (Agilent Technologies).

### Realtime PCR analysis of YAP mRNA levels

For the real-time PCR measurement of YAP RNA levels in the ESCC cells, approximately 106 of KYSE-450 cells were harvested from the Petri dish after ATO (10 µM) treatment for 24 h with a cell scrape stick, and suspended in 1 ml PBS. The cell suspensions were briefly centrifuged, and the pellet were resuspended in 1 ml Trizol (Invitrogen). Total cellular RNA was extracted by the Trizol Reagent according to the supplier’s instructions. cDNAs of the samples were synthesized with RevertAid First Strand cDNA synthesis Kit (Thermo Fisher). And real-time PCR amplification of the cDNAs were performed with SYBR^®^ Premix Ex Taq™ (TAKARA) kit in ABI 7500 real-time PCR system, with following specific primers:

**Table:** 

	Upper primer	Lower primer
YAP	5′ -ACTCGGCTTCAGCCATGAAC	3′ - AAGCCGTCCTCAATCGGGA
GAPDH	5′ -GAAGGTGAAGGTCGGAGT	3′ - CTTTAGGGTAGTGGTAGAAG

### Western blot and immunoprecipitation assay

Western blot experiments were performed with cell lysates harvested at different time points after ATO treatment. All the proteins were loaded into each well of a 15% SDS-PAGE after boiling. Gels were transferred onto PVDF membranes (Bio-Rad), blocked with 5% milk/PBS, and incubated overnight at 4 °C with primary antibodies. Following washing and incubation with secondary antibodies in 5% milk, the membrane was washed, and the positive signals were developed with chemiluminescence reagent (Amersham™). The immunoprecipitation assays were carried out with using Pierce™ IP lysis buffer (Thermo), protein A-agarose beads (Invitrogen), Ubiqapture-Q^®^ kit (ENZO life sciences). For immunoprecipitation, 2 µl Mouse anti-PML mAb, 20 µl of 50% slurry of protein G beads were added to each samples incubated at 4 ℃ for 16 h at a rotator in the immunoprecipitation experiment. Beads were washed four times with 1 ml of ten-fold diluted urea buffer and 20 µl of 2 × SDS loading buffer was added. Immunoprecipitations were analyzed by western blot as indicated. Antibodies: mouse anti PML antibody (Millipore Sigma, P6746), rabbit anti YAP antibody (CST, 14074), rabbit anti beta-actin antibody (Proteintech, 60008-1-Ig), HRP conjugated anti IgG secondary antibodies (Proteintech).

### Transwell migration and invasion assays

Corning’s 80 µm 24-well transwell plate coated with 30% Matrigel (300 µl/well, Falcon) was used for the migration and invasion assays were carried out with. 1 × 10^5^ cells in 100 µl of serum-free medium was added to each upper chamber of the transwell plate, and the lower chambers were filled with 600 µl of culture medium with 20% fetal bovine serum. The non-migrating cells remained in the upper chamber were removed from the upper surface of the Matrigel with a cotton swab after 6 h of incubation at 37 °C. The filters were fixed with methanol for 10 min, stained with Giemsa solution for 1 h, and counted. Five random microscopic fields (× 100) were counted per well, and the mean was determined.

### Apoptotic assay by flow cytometry

pCMV-Flag-YAP-5SA plasmid is generously provide by Bing Zhao’s lab of Zhejiang University. Five triplicated groups of KYSE-450 cells were seeded into 6-well plates at densities of 150,000 cells per well for 24 h. pCMV-Flag-YAP-5SA and empty pCMV vectors plasmid were transfected into KYSE-450 cells with Lipofectamine™ 3000 according to the manufacture’s protocol. The transfected KYSE-450 cells were further cultivated for 48 h to allow the over expression of YAP-5SA in the cells. Then three groups of the the YAP-5SA over expressing cells were treated with arsenic nano particle, AFCNC, and verteporfin respectively. 12 h after the treatments, the cells were collected by trypsinization. Apoptotic assay was performed with BD Pharmingen™ FITC-Annexin V Apoptosis Detection Kit and PI staining using Beckman Counter flow cytometer.

### Animal experiment

Protocols for the experiments involving animals were approved by the Animal Experimentation Ethics Committee at Zhejiang Cancer Hospital (Hang Zhou, China). Female BALB/c nude mice (4 weeks of age) were purchased from Hangzhou Hangsi Biotechnology Co., Ltd. These mice were housed in a pathogen-free animal facility of Zhejiang Cancer Hospital. The animal facility is registered in the National Animal Facility Registry which strictly adhered to the National Guidelines for Ethical Practice in animal experiments, Register ID SYXK2017-12.

AFCNC plus irradiation and cisplatin treatment in the ESCC mice xenograft model were carried out after the volumes of ESCC xenograft tumors reached 100 mm^3^ (at about 2 weeks after inoculation of 10^6^ KYSE-450 ESCC cells). AFCNC were administrated with intraperitoneal injection to the mice at a dose of 2.5 mg/kg every 6 days. Cisplatin (A8321, ApexBio. Ltd.) was administered to the mice with intraperitoneal injection at a dose of 5 mg/kg every 6 days. Before irradiation mice were anaesthetized with pentobarbital sodium by intraperitoneal injection at the dose of 75 mg/kg. Irradiation was administered to the mice xenografts zone at a dose of 5 Gy with SARRP small animal irradiator (Xstrahl Ltd. UK) at a dose rate of 4 Gy per minute using a range of custom collimators depending on the tumour geometry (220-kV, 13 mA X-ray, 10mm × 10 mm).

### Immunohistochemistry experiment

ESCC xenograft tumors were separated from the mouse after sacrifice, dissected tumors were fixed in 4% neutralized formaldehyde, embedded in paraffin. Blocks of paraffin-embedded donor tissue were sampled using a Manual Tissue Arrayer instrument (Beecher Instruments, Silver Spring, MA, USA). A serial of 4-µm-thick sections were cut for the purpose of immunohistochemistry and transferred to adhesive slides according to manufacturer’s instructions.

Standard IHC analysis was performed with the primary antibodies against human Cox-2 (Proteintech 12375-1-AP), YAP (13584-1-AP) at a dilution in 1:100. In brief, the tissue microarray slides were deparaffinized in xylene and gradient ethanol. Antigen retrieval was performed by placing the slides in a high-pressure cooker in a 0.01 mM citrate buffer, pH 6.0, for 2.5 min at 100 °C; they were then cooled for 20 min. Endogenous peroxidase activity was blocked by incubating the section in 3% H_2_O_2_ for 10 min, followed by rinsing in PBS solution three times, tissue microarray slides were preincubated with blocking serum and then were incubated with the primary antibodies at 4 ℃ overnight. After three washes in PBS, the slides were treated with the horseradish peroxidase (HRP)-labeled goat anti rabbit secondary antibody (Dako, Glostrup, Denmark) for 50 min at room temperature. After washing with PBS, reaction products were visualized with 3, 3′-diaminobenzidine (DAB, Dako, Glostrup, Denmark) and the slides were counterstained with hematoxylin. After being dehydrated, slides were mounted in resin. Immunohistochemistry results were evaluated by scanning each slide under low power magnification (× 100) to identify regions containing positive immunoreactivity.

## Results

### Preparation of arsenic–ferrosoferric oxide (As_2_S_2_–Fe_3_O_4_) conjugated Nano Complex (AFCNC)

**Fig. 1:**
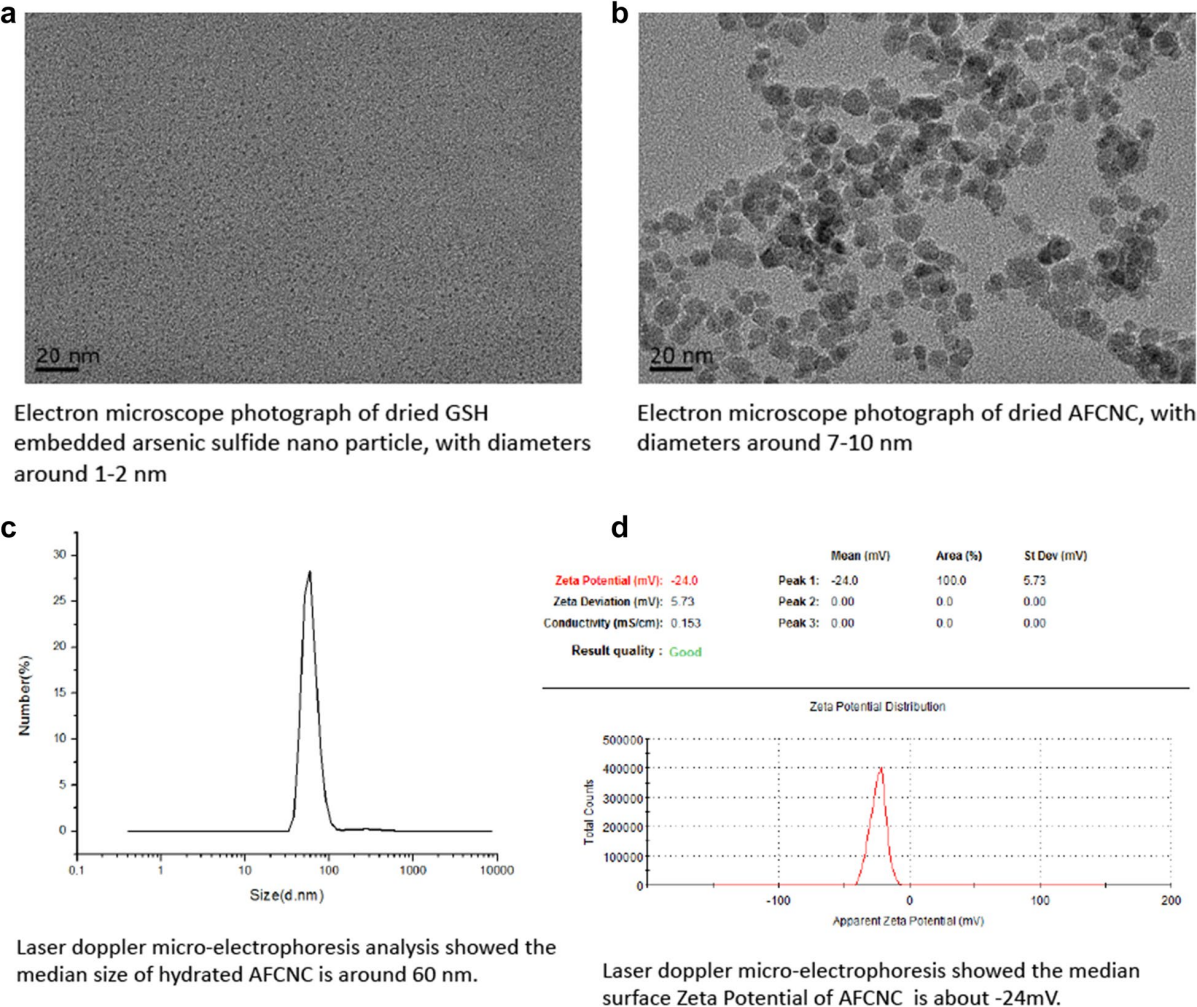

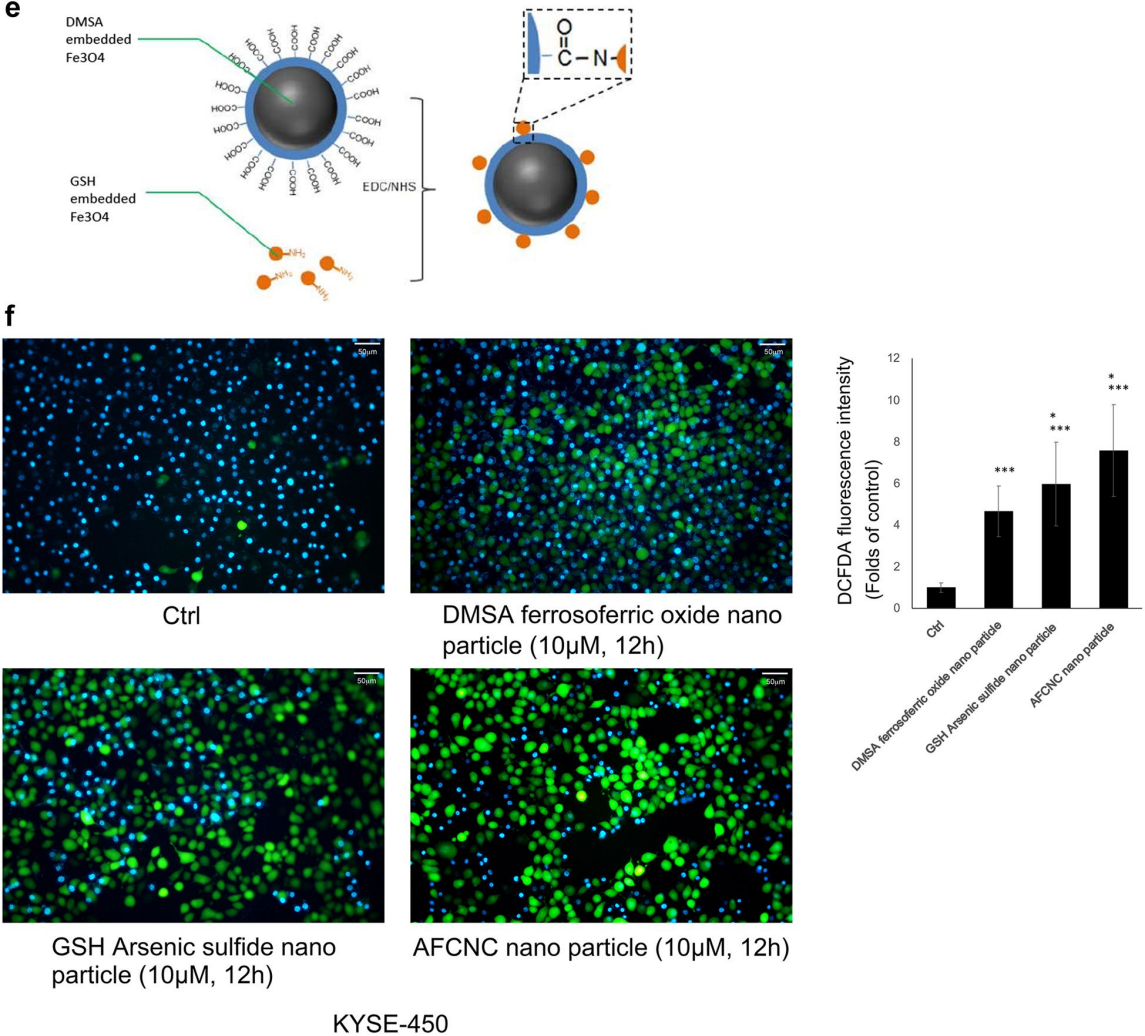
Physical and ROS induction properties of AFCNC

GSH embedded arsenic sulfide nano particle and DMSA embedded ferrosoferric oxide nano particle was prepared as described in "Materials and methods" section. Electronic microscope observation of dried GSH embedded arsenic sulfide nano particles showed with diameters around 1–2 nm (Fig. 1a). We dissolved 10 mg DMSA embedded ferrosoferric oxide nano particle into 10 ml PBS solution, and adjust the pH value to 6. Then we added 20 mg GSH embedded arsenic sulfide nano particle to the DMSA embedded ferrosoferric oxide suspension, and incubated for 30 min. 10 mg of EDC (1-ethyl-3-(3-dimethylaminopropyl) carbodiimide hydrochloride) was added to the suspension at room temperature for 24 h to promote amide formation (Fig. 1e). The acquired As_2_S_2_–Fe_3_O_4_ conjugated nano complex was harvested with a magnet. Electronic microscope observation of dried the AFCNC nano complex showed diameters of 7–10 nm. (Fig. 1b) The zeta potential and electrophoretic mobility of AFCNC was measured with laser doppler micro-electrophoresis, which showed AFCNC has a median hydrated diameter around 60 nm, and a zeta potential of -24 mV (Fig. 1c, d). These results suggested AFCNC forms a relatively stable suspension in water.

Then we employed DCFH-DA cellular ROS detection assay to evaluate the ROS induction ability of AFCNC in KYSE-450 cells. Upon 12 h of treatment AFCNC, KYSE-450 cells showed with significantly brighter green fluorescent staining compared with treatment with DMSA ferrosoferric oxide nano particle or GSH arsenic sulfide nano particle (Fig. 1f), which indicated stronger ROS induction ability of AFCNC.

### Hippo signaling pathway is affected by arsenic treatment

**Fig. 2:**
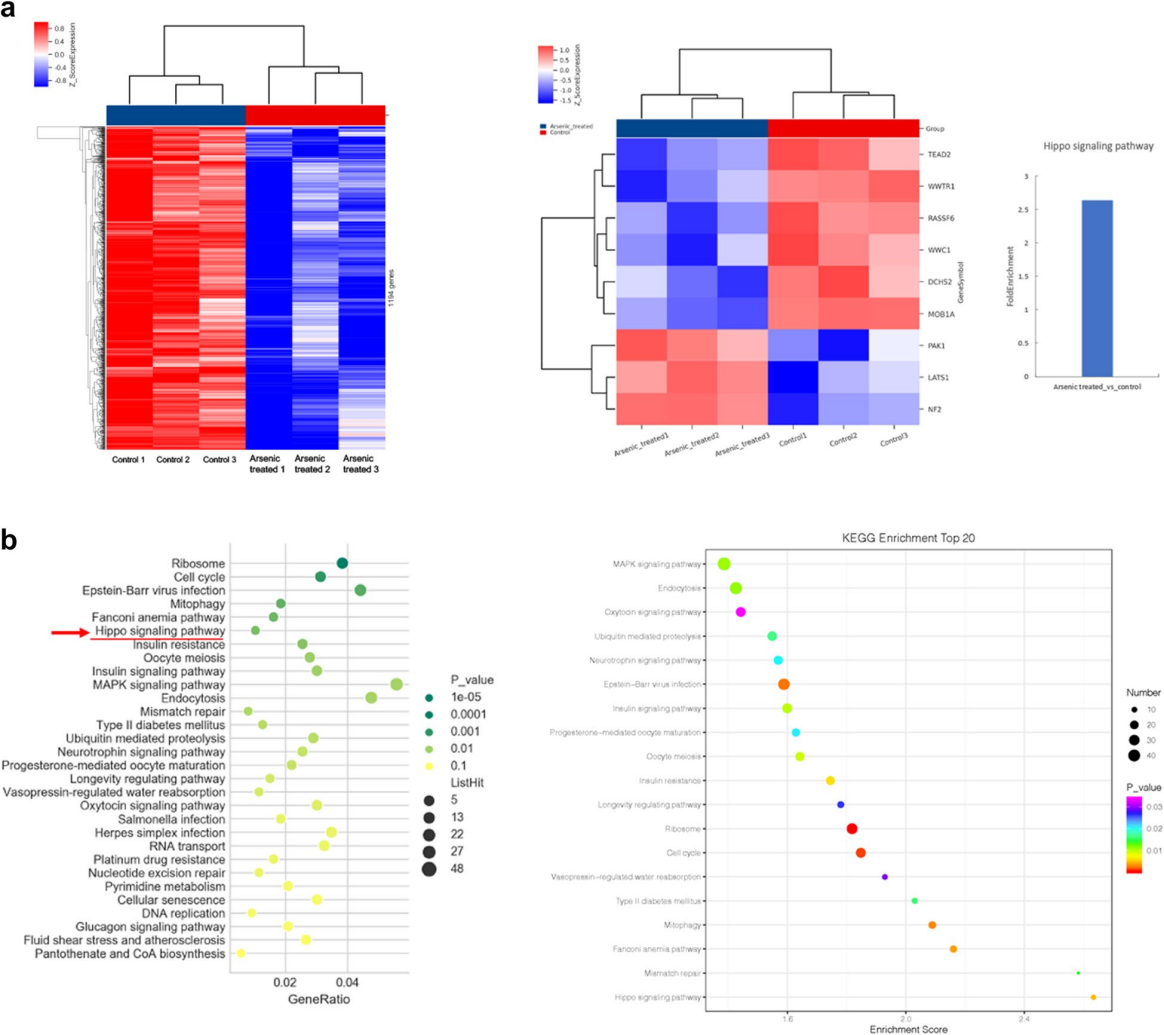
Gene expression microarray analysis of pathways affected by Arsenic treatment

To screen for the molecular pathways that are affected by arsenic treatment, we treated KYSE-450 cells with 10 µM of GSH embedded arsenic sulfide nano particle for 24 h. The cells were harvested, and the total cellular RNA transcripts were extracted to construct the cDNA libraries of ESCC cancer cells. The cDNA libraries were subjected to Agilent Human lncRNA Microarray analysis. We found Hippo signaling pathway is strongly affected by arsenic sulfide treatment, which has the highest enrichment score among all the signaling pathways analyzed in the gene microarray, and the p value of the differentially expressed genes affected by arsenic treatment in the Hippo pathway is 0.0047. (Fig. 2a, b). In particular, we found arsenic sulfide nano particle treatment significantly reduced genes transcripts that are regulated by YAP, the key transcription factors of the Hippo signaling pathway (Fig. 2a).

### Arsenic treatment induced degradation of YAP

**Fig. 3:**
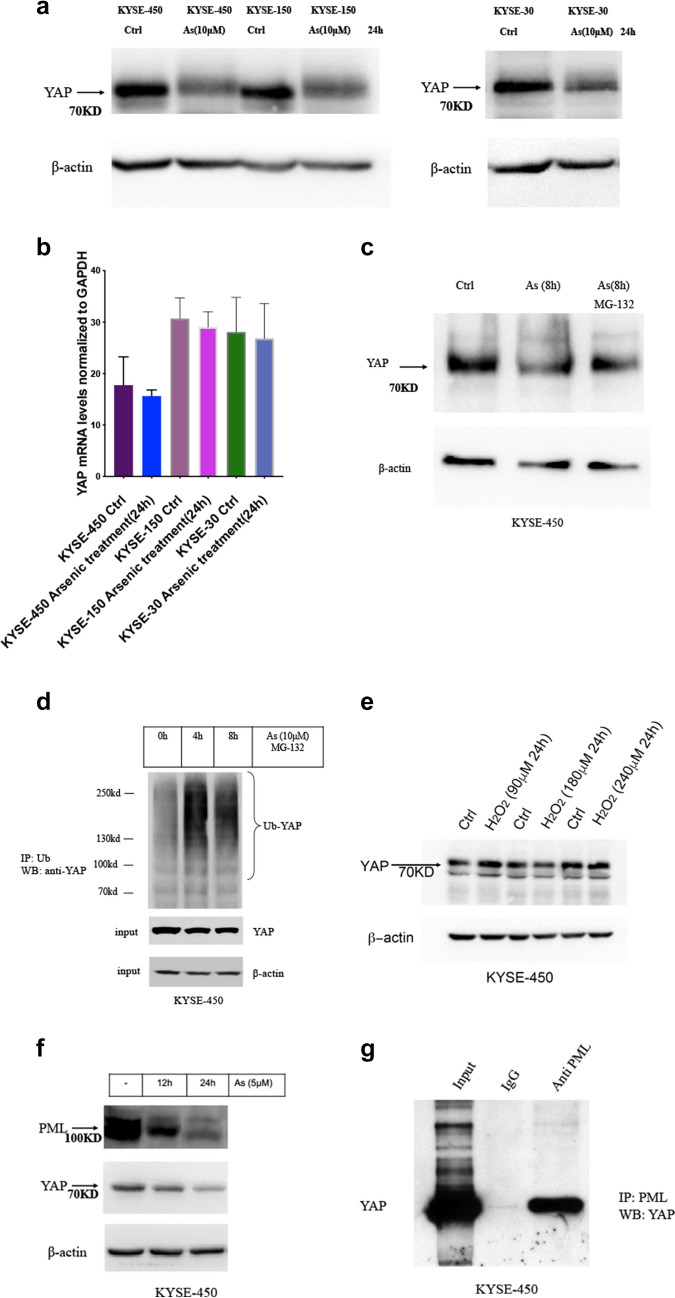
Molecular studies of arsenic induced degradations of YAP

We hypothesized YAP may be another target for arsenic compound in cancer cells. With western blot assay we found 24 h treatment with arsenic nano particle induced significant decreases of YAP protein levels in ESCC cancer cells (Fig. 3a). Whereas real time PCR analysis showed arsenic treatment did not significantly alter the mRNA levels of YAP (Fig. 3b). And proteasome inhibitor MG-132 partially inhibited arsenic compound induced degradation of YAP (Fig. 3c). With protein immunoprecipitation assay we found Arsenic treatment induced a significantly increased ubiquitinated YAP (Fig. 3d). Together these findings suggested arsenic treatment induced a significant increase of ubiquitin mediated proteasomal degradation of YAP. Arsenic compounds are known to induced ROS in cancer cells [8]. To test if degradation of YAP is caused by the ROS responses in ESCC cancer cells, we treated KYSE-450 cells with hydrogen peroxide(90 µM–270 µM) for 24 h. We observed no significant changes of YAP protein levels by hydrogen peroxide treatment with western assay (Fig. 3e).

Because YAP protein does not contain a cysteine rich motif (CCXXCC) that specifically binds to arsenic compound, YAP is not likely to bind to arsenic compound directly. Arsenic compound is known to directly binds to PML containing protein complexes in the cell to promote their proteasomal degradations [3]. And arsenic compounds causes ubiquitination mediated degradation of PML containing molecular species in cancer cells [17]. Consistently, with western blot assay we observed arsenic treatment in ESCC KYSE450 cells induced degradation of PML, concurred with the degradation of YAP (Fig. 3f). Lapi et al. reported PML physically interacts with YAP and inhibited ubiquitination mediated degradation of YAP by promoting sumoylation of the YAP protein [18]. We speculated that arsenic induced degradation of YAP is mediated by arsenic induced degradation of PML. With immunoprecipitation assay, we found YAP physically interacted with PML in ESCC KYSE-450 cancer cells (Fig. 3g).

### Arsenic treatment significantly reduced the migration and invasion of ESCC cancer cells

**Fig. 4:**
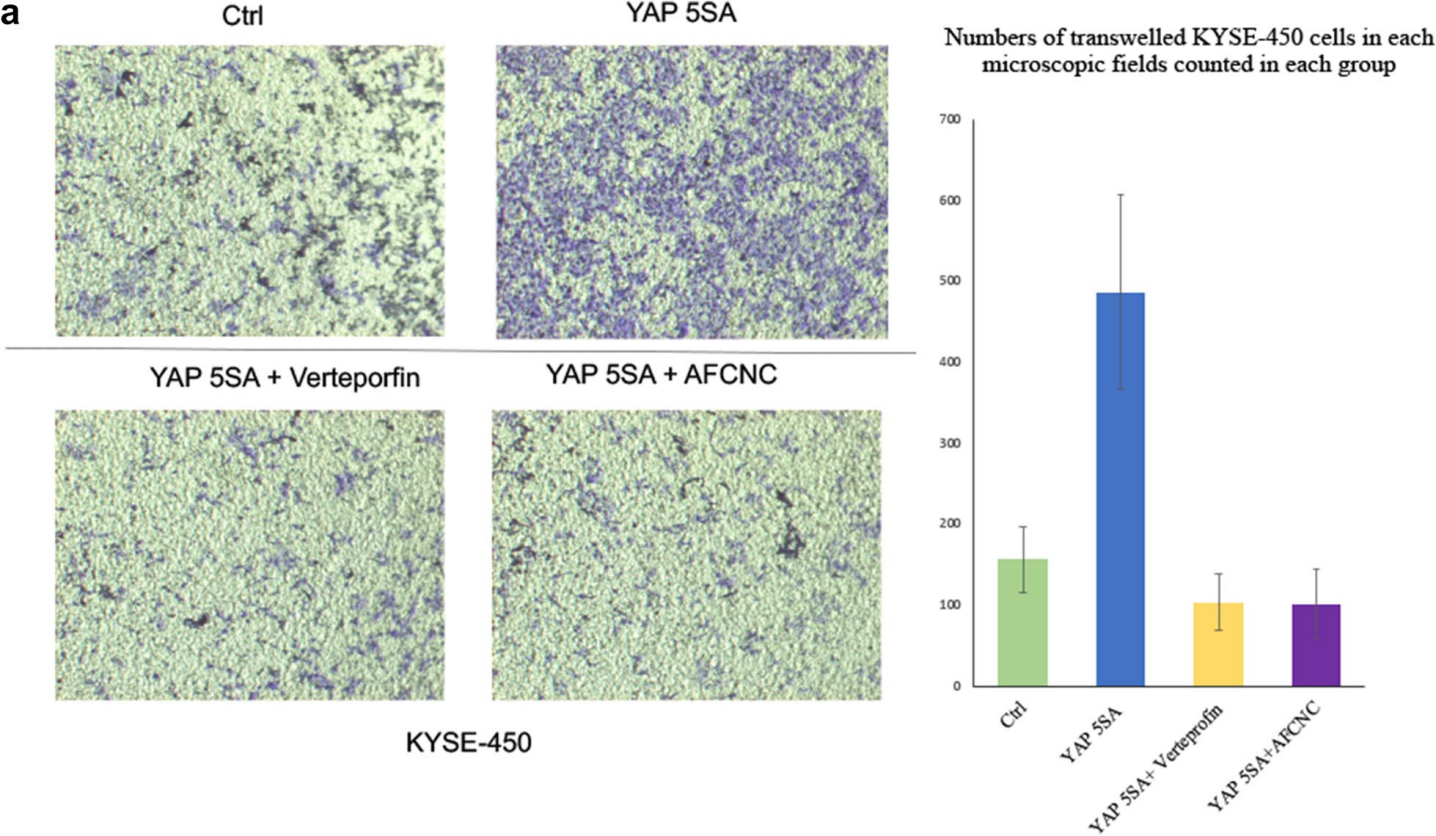

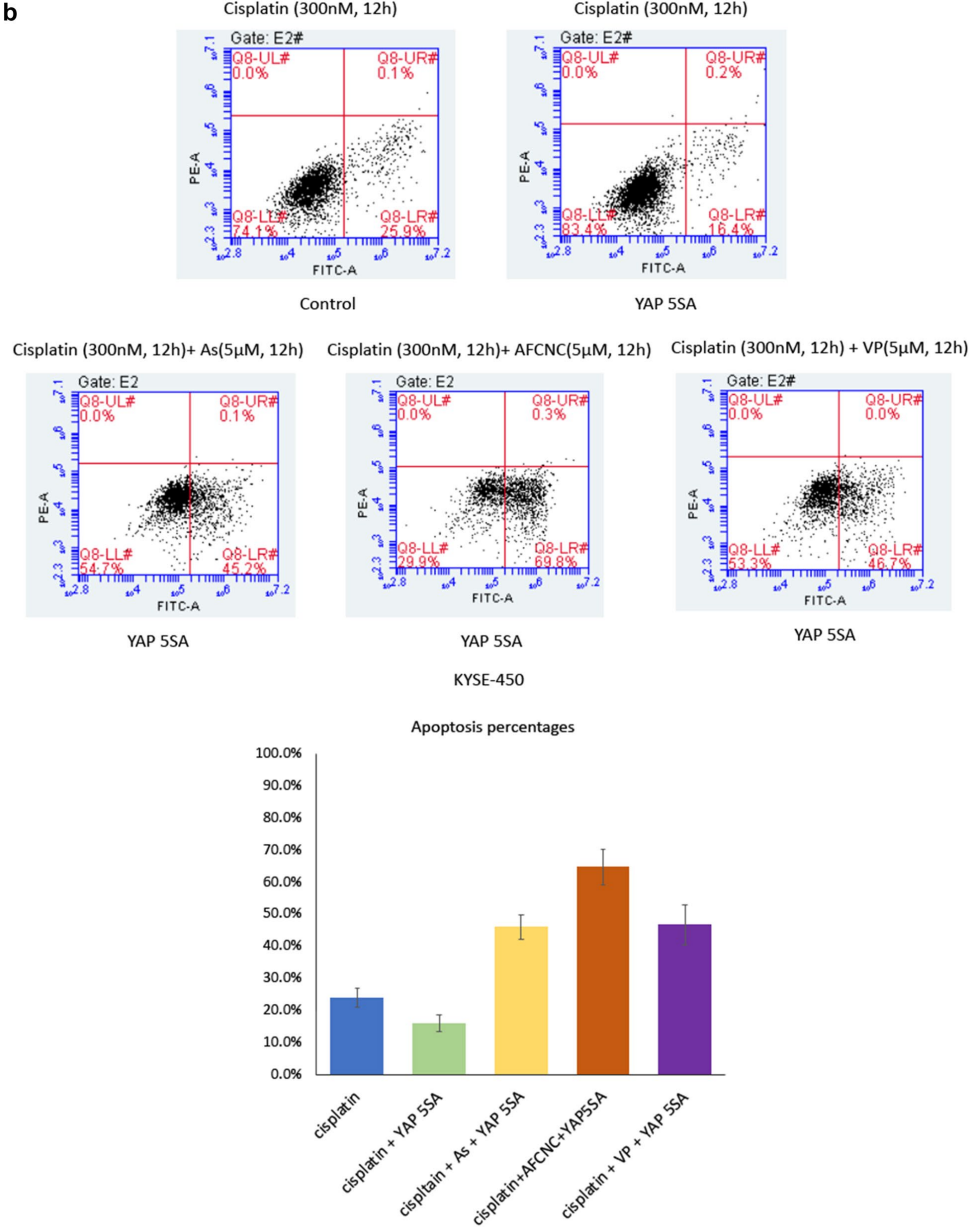
Effects of YAP and AFCNC on cancer cell migration and apoptosis

Hippo signaling pathway transcriptional factor Yap is known to regulate the invasion and migration of esophageal cancer cells [14]. We found overexpression of a constitutive active form of YAP (YAP5SA) significantly increased the invasion and migration ability of KYSE-450 cells in the transwell assay. Whereas, YAP-Tead transcriptional inhibitor Vertepofin or arsenic treatment significantly decreased invasion and migration of KYSE-450 cells overexpressing YAP5SA in the transwell experiment (Fig. 4a).

### Arsenic treatment increased cisplatin induced apoptosis of YAP5SA overexpressed ESCC cancer cells

Overexpression of YAP increased cancer stem cell populations and induced multiple drug resistances in esophageal cancer and several other types of cancers [14, 16, 19, 20]. Consistently, we found overexpression of YAP5SA in KYSE-450 cells significantly reduced apoptosis induced by cisplatin treatment in the flowcytometry analysis. Whereas treatment of arsenic sulfide nano particle or AFCNC significantly increased the apoptotic cells population in ESCC cancer cells overexpressing YAP5SA with a similar effect to the treatment of YAP transcriptional inhibitor Verteporfin (Fig. 4b). Together, these findings suggested YAP overexpression induced drug resistance could be inhibited by YAP inhibitor or arsenic treatment in ESCC cancer cells.

### Intraperitoneal injection AFCNC significantly inhibited tumor growth in combination with cisplatin or radiation in ESCC mouse xenograft model

**Fig. 5:**
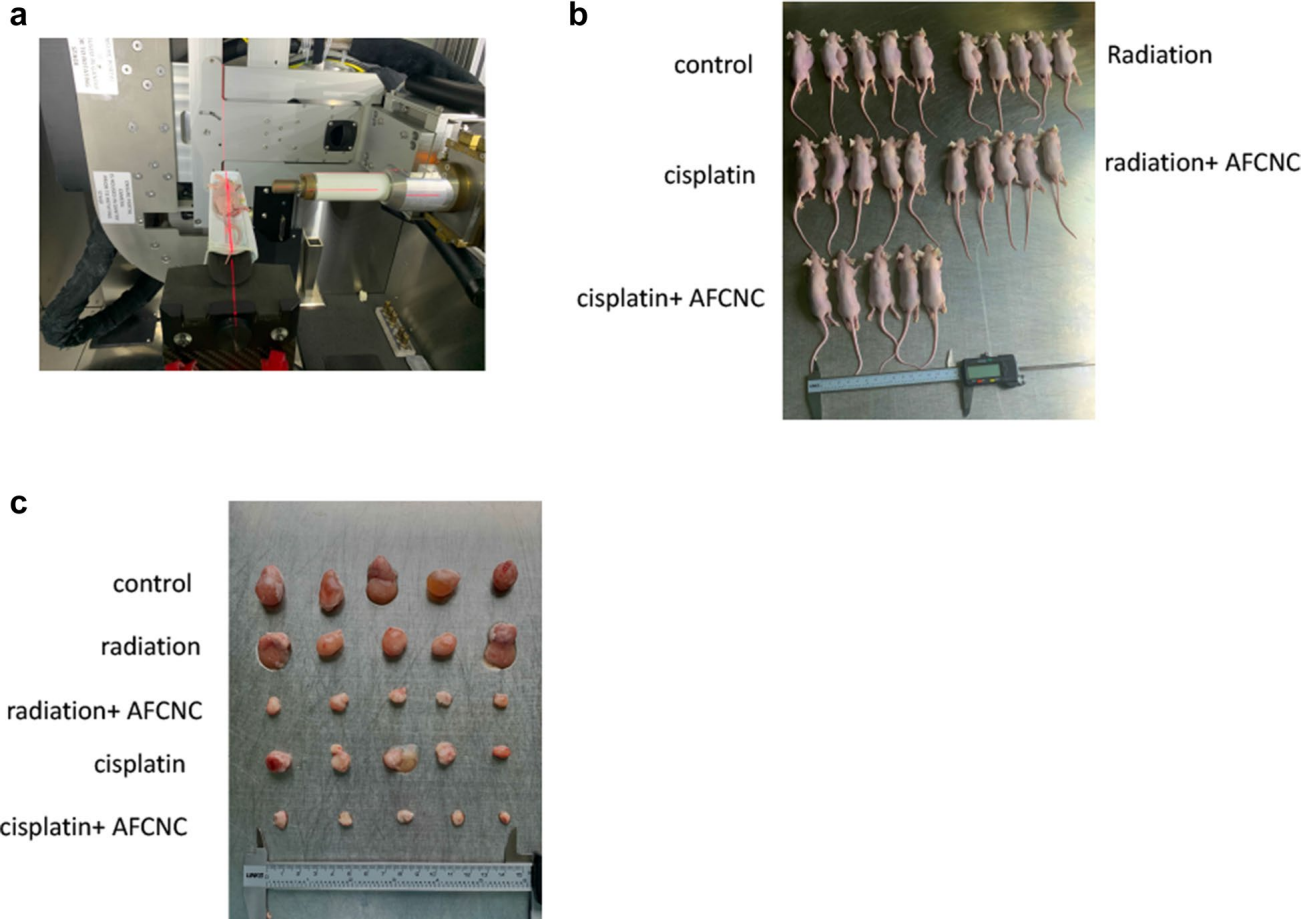

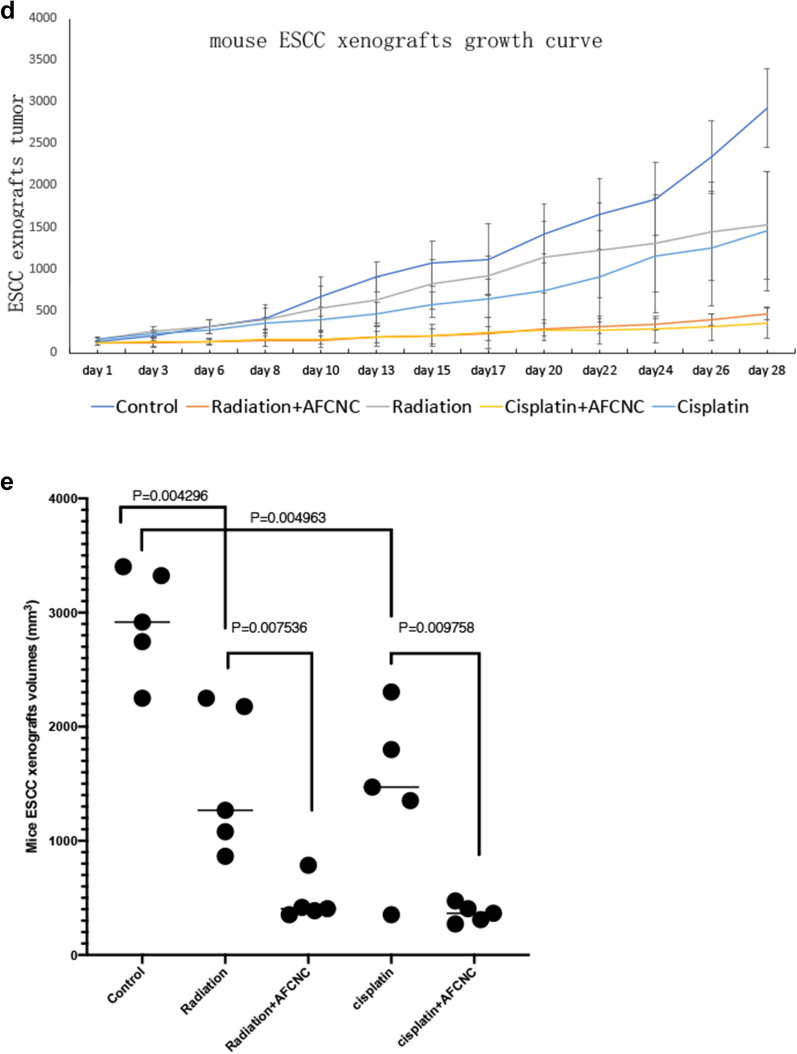
ESCC mouse xenograft experiment studying the synthetic effects of AFCNC with Chemo and radiation therapy

Because YAP is known to be involved in chemo and radiation resistances in ESCC, we used nude mouse xenograft model to study the tumor inhibitory effects of AFCNC in combination with Radiation therapy and cisplatin treatment in vivo. We observed combinatory administration of AFCNC with cisplatin or after radiation therapy significantly reduced the growth rates and tumor volumes of the KYSE-450 ESCC xenograft tumors in nude mice compared with cisplatin treatment or radiation alone, without observable side effects (Fig. 5a–e).

**Fig. 6:**
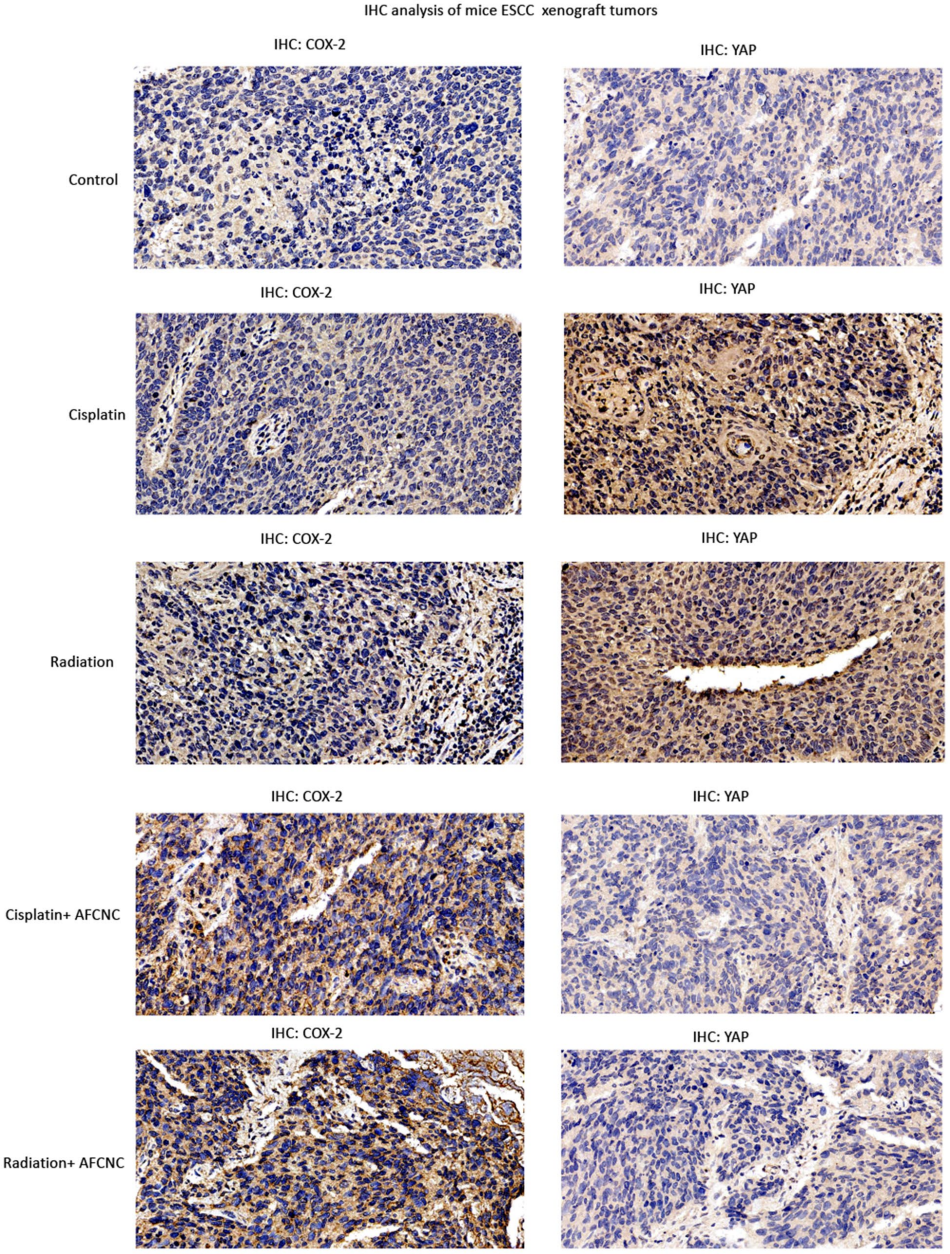
IHC analysis of ESCC mouse xenografts

Lastly, immunohistochemistry assays were performed to analyze the COX-2 and YAP expression levels in the ESCC xenografts of each treatment groups. We observed AFCNC treatment significantly increased the COX-2 expression levels in ESCC xenografts, which is known to induced by ROS in the tumor tissues [21]. Cisplatin and radiation treatments significantly increased the expression levels of YAP in ESCC xenograft tumors. Whereas combinatory treatments of AFCNC significantly reversed the increased YAP expression levels induced by cisplatin and radiation in the ESCC xenograft tumors (Fig. 6). Collectively, these findings suggested combined administration of AFCNC sensitized ESCC cancer cells to radiation therapy and cisplatin based chemotherapy in the mice xenograft model. And besides ROS, YAP is likely to be another important molecular target in mediating the synthetic effects of arsenic reagent with chemo and radiation therapy.

## Discussion

Esophageal cancer is one of the leading causes of death among all cancer patients in China [12]. Animal studies suggested intratumoral delivery arsenic compound efficiently suppresses cancer growth in ESCC mouse xenograft model without systemic side effects [22]. Despite of failures of using arsenic trioxide as a single agent in the treatment of esophageal cancer, arsenic compounds are still promising to be used as a combinatory chemotherapeutic drug to increase the chemo and radio sensitivity of solid tumors [8]. In this study, we used gene transcriptomic microarray to screen for the signaling pathways that are most affected by arsenic treatment in ESCC cancer cells, and we identify Hippo signaling pathway is among the top hits of pathways affected by arsenic treatment in ESCC cancer cells. We further proved arsenic treatment induced ubiquitination mediated degradation of YAP in esophageal cancer cells. Lapi et al. reported PML physically interacts with YAP to promote its stability in cancer cells [18]. We proved arsenic induced degradation of PML and YAP in KYSE-150 cells, and YAP physically interacted with PML in KYSE-450 cancer cells. Thus, arsenic treatment induced degradation of YAP is likely to be mediated by the degradation of PML in ESCC cancer cells.

Hippo signaling pathway is frequently mutated in Esophageal squamous cell carcinoma (ESCC), Song et al. reported Hippo transcription factor YAP and its upstream regulatory elements are mutated in 44% of ESCC cells [23]. Wang et al. reported YAP positively regulates SOX9 and confers cancer stem cell features of ESCC cells [14]. Consistently, in this study we found overexpression of the transcriptional active form of YAP(YAP5SA) in ESCC cancer cells increased migration and invasion ability of ESCC cancer cells, and reduced apoptosis induced by cisplatin treatment. Whereas treatment with arsenic nano particles or YAP specific inhibitor Verteporfin reversed the effects of YAP5SA overexpression, which significantly decrease the invasion and migration ability of YAP5SA overexpressing ESCC KYSE-450 cells and increased the apoptotic population of KYSE-450 cells induced by cisplatin.

Zanganeh et al. reported iron oxide nanoparticles promote Th1 polarization of macrophages in the tumor microenvironment and inhibited tumor growth through the production of ROS species [24]. Shen et al. reported iron oxide-cisplatin conjugated nano particles induced more dramatic ferroptosis in brain tumor cells though accelerating Fenton reaction [11]. These studies suggested iron oxide nano complex alone or in combination with other materials could be used as a supplement to increase the tumor killing efficacies of classic chemotherapeutic drugs. In this study we developed arsenic–ferrosoferric oxide conjugated nano complex (AFCNC) to promote the ROS inducing potential arsenic sulfide nano particle by promoting Fenton reaction [11]. Laser doppler micro-electrophoresis assay showed AFCNC with an average hydrated diameter around 60 nm is stable in water as indicated by the surface Zeta potential. We found AFCNC is a more potent inducer of ROS compared with unconjugated arsenic or ferrosoferric oxide nano particles in KYSE-450 cells with DCFH-DA ROS detection assay. And it is more potent to promote cisplatin induced apoptosis in KYSE-450 cells against YAP5SA overexpression. With ESCC mouse xenograft model we further proved AFCNC is an effective arsenic nano reagent to sensitize ESCC cancer cells to radiation and chemotherapy in vivo.

Hu et al. reported transcatheter arterial chemoembolization plus an intravenous infusion of arsenic trioxide effectively controlled pulmonary metastasis and prolonged overall survival in patients with HCC [4]. Whereas, Hayashi et al. reported overexpression of Hippo transcription factor YAP/TAZ in hepatocellular carcinoma (HCC) is associated with chemoresistance and poor prognosis [25]. Our findings suggested YAP may be an effective molecular target for arsenic treatment of HCC.

Several other studies indicated YAP functions as supper enhancer binding factor that regulates transcriptional pause release in the cells [26, 27]. Cai et al. and Lu et al. reported YAP and TAZ bound to super enhancers to initiate liquid-liquid phase separation of chromatin condensates to facilitate the transcriptional programing of RNA polymerase II [28, 29]. Kurppa et al. reported YAP activation is essential for EGFR/MEK inhibition induced tumor dormancy and apoptotic reprogramming by suppressing of BMF, and by targeting YAP-TEAD transcription complex offered an unique treatment strategy for combined therapies to overcome drug resistance and tumor relapses [16]. YAP and TAZ also transcriptional upregulate the expression of immune checkpoint protein PD-L1 on cancer cells to suppress the antitumor effects of T cells [30, 31]. YAP inhibitors are promising drug candidates for suppressing cancer relapses and tumor immune suppression, and several YAP inhibitors have been developed [32]. Our finding that arsenic compound induced degradation of YAP in ESCC cancer cells should have a general application in arsenic based therapeutics in other types of tumors, and could be used as an compound to inhibit YAP signaling for synthetic therapeutics against tumor immune evasion and drug resistance of other targeted inhibitors.

## Conclusions

Arsenic compounds are promising adjuvant drugs to be used to sensitize solid tumors to chemo and radiation therapy. In this study we developed arsenic–ferrosoferric oxide (As_2_S_2_–Fe_3_O_4_) conjugated Nano Complex which showed with increased ROS induction ability in ESCC cancer cells. We identified Hippo signaling pathway transcription factor YAP as a molecular target for arsenic induced synthetic effects with radiation and cisplatin treatment in ESCC cancer cells in vitro and in vivo. These findings suggested arsenic compounds could be used as an adjuvant therapeutics for chemo and radiation therapy to inhibit treatments induced cancer stem cell features and drug resistance mediated by YAP in solid tumors.
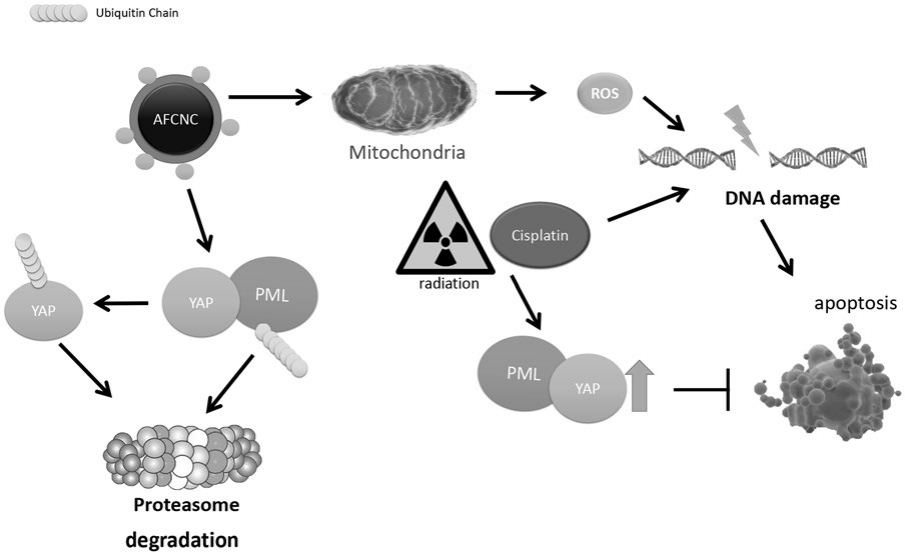


## Data Availability

Not applicable.

## Abbreviations

ROS: Reactive oxygen species
GSH: Glutathione
DMSA: Dimercaptosuccinic acid.
AFCNC: Arsenic–ferrosoferric oxide conjugated Nano Complex
ESCC: Eesophageal squamous cell carcinoma
DCFH-DA: 2,7-Dichlorodi -hydrofluorescein diacetate
IHC: Immunohistochemistry
