# Correction to: Arsenic nano complex induced degradation of YAP sensitized ESCC cancer cells to radiation and chemotherapy

**DOI:** 10.1186/s13578-021-00535-2

**Published:** 2021-01-19

**Authors:** Wei Zhou, Meiyue Liu, Xia Li, Peng Zhang, Jiong Li, Yue Zhao, Guogui Sun, Weimin Mao

**Affiliations:** 1 Cancer Hospital of University of Chinese Academy of Sciences (Zhejiang Cancer Hospital), Institute of Cancer and Basic Medicine of Chinese Academy of Sciences, Hangzhou, 310022 China; 2 North China University of Science and Technology Affiliated Peoples Hospital, School of Public Health, North China University of Science and Technology, Tangshan, 063001 China; 3 Department of Medicinal Chemistry, Massey Cancer Center, Philips Institute for Oral Health Research, Virginia Commonwealth University, Richmond, Virginia 23298-0540 United States

## Correction to: Cell Biosci (2020) 10(1): 1−5 10.1186/s13578-020-00508-x

Following publication of the original article [1], the affiliation of the first two authors was incorrect. The affiliations are presented correctly in this correction article.

The original article has been corrected.
